# Trends in Knee and Hip Arthroplasty in Chile Between 2004 and 2019

**DOI:** 10.7759/cureus.12185

**Published:** 2020-12-20

**Authors:** Maximiliano Barahona, Cristian Barrientos, Francisco Escobar, Nicolas Diaz, Daniel Palma, Macarena A Barahona, Alvaro Martinez, Carlos A Infante

**Affiliations:** 1 Orthopaedic Department, Hospital Clinico Universidad De Chile, Santiago, CHL; 2 Orthopaedic Department, Hospital Clinico Universidad de Chile, Santiago, CHL

**Keywords:** knee arthroplasty, hip arthroplasty, national registries, arthroplasty, epidemiology

## Abstract

Introduction

The purpose of this study is to describe the incidence rate (IR) per 100,000 inhabitants of arthroplasty in Chile between 2004 and 2019, emphasizing knee and hip arthroplasty.

Methods

This is a cross-sectional study. Patients who underwent arthroplasty between 2004 and 2019 were identified in the free access database of the Chilean Department of Statistics and Health Information (DEIS), which depends directly on the Ministry of Health. This register stores all hospital discharges of the country from private or public health centers. The trend during the period of study was analyzed using Spearman's correlation.

Results

From a total of 111,303 patients, 133,518 arthroplasties were performed. Hip arthroplasty (HA) accounted for 73.35%, followed by knee arthroplasty (KA) (23,92%). A significant upward trend was found in HA (rho=0.95, p<0.000) and KA (rho=0.98, p<0.000). Most of the surgeries were done within the Public Health Network (61,6%), but 20% of patients affiliated with public insurance underwent arthroplasty in a private center. Patients above 60 years of age affiliated with private insurance underwent 1.8 HA and 2.5 KA for every one HA and KA undergone by patients of the same age group who were affiliated with public insurance.

Conclusion

HA was more frequent than KA. A significant gap was found in the incidence of arthroplasty as compared to countries belonging to the Organization for Cooperation and Economic Development, given by a less aged population and by inequity in health access. Wider coverage and a national registry for arthroplasty must be considered in Chilean health policies.

## Introduction

There are 40 arthroplasty registries worldwide, including countries of Europe, North America, Africa, Asia, and Oceania. In contrast, Chile, along with other Latin American countries, have not developed registries. Brazil is the only one that has reported a project to be implemented in the near future [1].

A recent study carried out by the Organization for Cooperation and Economic Development (OECD) estimates that the number of hip arthroplasties within the next 35 years will grow 100%, overstressing health systems [2]. National registries offer the potential to better outcomes and identify, implement, and share best practices. The effective use of quality registries can lead to better health outcomes at a lower cost for society [3].

In the Chilean health system, population coverage is provided mainly by two insurances: the public by the National Health Found (FONASA) and the private by the Health Insurance Institutions (ISAPRE). During the period studied, 72.5% of the Chilean population was affiliated with FONASA; meanwhile, 17.4% was affiliated with ISAPRE.

Two types of institutions provide inpatients services: the public health network (PHN) and private health institutions (PHI). PHN attends patients affiliated with FONASA. PHI corresponds to a broad spectrum that includes university hospitals, workers' mutuals, armed forces hospitals, police hospitals, and centers belonging to investment funds. Some patients affiliated with FONASA choose treatment in PHI at their expense to avoid the wait time for surgery.

The purpose of this study is to describe the incidence rate per 100,000 inhabitants (IR) of arthroplasty in Chile, emphasizing knee and hip arthroplasty. The hypothesis is that hip and knee arthroplasty have an upward trend, the IR of hip arthroplasty being higher than knee arthroplasty.

## Materials and methods

**Figure 1:**
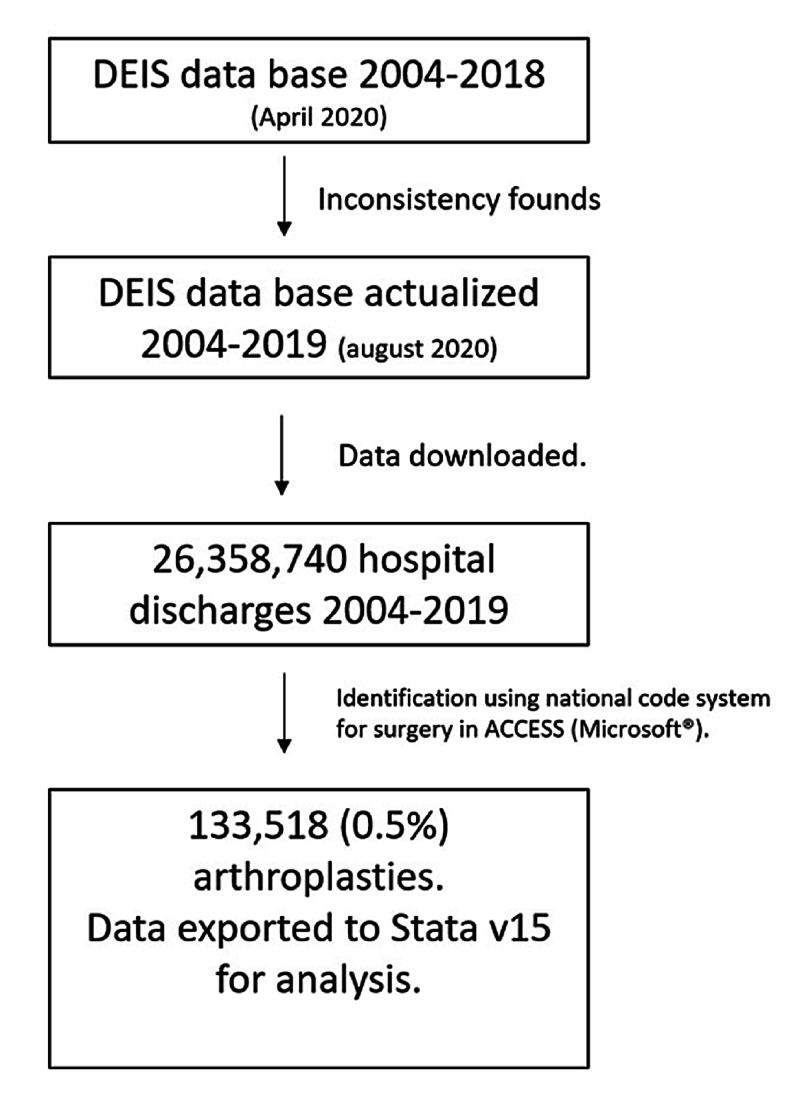
Flowchart DEIS: Department of Statistics and Health Information

This is a cross-sectional study. Patients who underwent arthroplasty between 2004 and 2019 were identified in the free access database of the Chilean Department of Statistics and Health Information (DEIS), which depends directly on the Ministry of Health. This register stores all hospital discharges of the country, both from PHN and PHI. During the analysis of the database, inconsistencies were found in the years 2006 and 2010 to 2012. This was reported to DEIS via the National Transparency Portal in April 2020. The last database updates of each of those years were used. Registres from 2006 were updated on August 11, 2020, and the ones between 2010 and 2012 on May 22, 2020 (Figure 1).

The databases from 2004 to 2019 were downloaded from the DEIS homepage https://deis.minsal.cl/#datosabiertos. The software program Microsoft Access (Microsoft Corporation, Redmond, Washington) was used to manage the data. Patients were identified using the national code for surgeries: hip arthroplasty (HA) = 2104128, 2104129 2104228 and 2104229; knee arthroplasty (KA) = 2104153; shoulder arthroplasty (SA) = 2104042 and 2501044; elbow arthroplasty (EA) = 2104062; wrist arthroplasty (WA) = 2104179; ankle arthroplasty (AA) = 2104174. Data were exported to STATA v.15 (StataCorp LP, College Station, Texas) for statistical analysis.

The incidence rate (IR) was calculated per 100,000 inhabitants using the overall population published in the annual report of the National Statistics Institute of Chile (INE) [4]. From the same report, the number of men and women was used to calculate the IR per gender. The proportion of patients affiliated with public insurance (FONASA) or private insurance (ISAPRE) was obtained from the annual report of FONASA and was used to calculate the IR per health coverage. Additionally, in each health insurance, the IR of patients older than 60 years of age - who are at a higher risk of needing an arthroplasty - was calculated.

The data has the primary diagnosis coded according to the International Classification of Diseases, 10th revision (ICD-10). Patients undergoing arthroplasty due to fractures were identified according to the ICD-10 classification. It was not possible to recognize if the patient underwent unicompartmental, patellofemoral, or total knee replacement, as all those procedures have the code 2014153. Also, no different code is used to identify if the prosthesis was constrained or unconstrained, primary, or revision. In the case of hip replacement, it was possible to detect total and hemiarthroplasty, as they have different codes. The revision rate was estimated, including all patients who had more than two arthroplasties in the same joint and patients diagnosed with mechanical complications or infections.

Spearman’s correlation was used to analyze the trend in the period studied; a significance of 0.05 was used. If the trend was significant, a linear regression was estimated to predict the IR in 2030. The r-squared (R2) and parameters β0 and β1 are reported. After every linear regression, heteroscedasticity and the normal distribution of the residuals were evaluated.

## Results

A total of 133,518 arthroplasties were found, which represents 0,5% of the hospital discharges between 2004 and 2019; the PHN managed 82,287 (61.6%) surgeries. Procedures in patients affiliated with FONASA were 97,065 (72.60%), and in patients affiliated with ISAPRE were 23,948 (17.9%). The number of patients included was 111,303, with a mean age of 67 years (±14.44), and 72360 (65.01%) were female.

The most frequent arthroplasty was HA 97,931 (73.35%), followed by KA 31,943 (23,92%), SE 2,735 (2.05%) AA 369 (0.28%), WA 284 (0.21%), and EA 256 (0.19%). The IR of each arthroplasty is shown in Table 1.

**Table 1:** Rate of arthroplasty per 100,000 inhabitants per year Abbreviations: HA: hip arthroplasty; KA: knee arthroplasty; SA: shoulder arthroplasty; EA: elbow arthroplasty; AA: ankle arthroplasty; WA: wrist arthroplasty

Year	HA	KA	sa	ea	aa	wa
2004	13.18	2.56	0.14	0	0.01	0.02
2005	17.15	2.59	0.29	0.01	0.01	0.27
2006	18.19	3.61	0.22	0.01	0	0.05
2007	24.05	5.22	0.42	0.04	0.02	0.11
2008	24.32	5.99	0.44	0.04	0.06	0.13
2009	29.58	7.41	0.54	0.05	0.10	0.05
2010	28.21	6.41	0.54	0.03	0.11	0.09
2011	43.71	7.98	0.59	0.10	0.15	0.13
2012	38.17	10.77	0.84	0.07	0.08	0.07
2013	37.99	13.91	1.17	0.18	0.12	0.10
2014	37.56	10.29	1.29	0.11	0.12	0.09
2015	42.39	14.33	1.30	0.12	0.07	0.09
2016	45.50	16.59	1.61	0.13	0.27	0.17
2017	50.78	18.84	1.86	0.11	0.44	0.10
2018	49.58	23.84	2.01	0.22	0.24	0.08
2019	54.49	28.23	2.01	0.20	0.24	0.14

Hip arthroplasty

A total of 97,931 HA were found; of them, 63,323 (64.66%) were performed in the PHN. The median age was 67 years (±15) and 41,325 (42.20%) patients were under 65 years of age. Procedures on patients affiliated with FONASA were 73,307 (74.86%); meanwhile, 16,249 (15.59%) were affiliated with ISAPRE. In 2004, the IR of HA was 13.18, which increased to 54.49 in 2019. This upward trend was significant (rho=0.95, p<0.000) (Table 2).

**Table 2:** Summary of the findings of hip arthroplasty *Per 100,000 inhabitants Abbreviations: HA: hip arthroplasty; THA: total hip arthroplasty; fracture: hip replacement due to femoral neck fracture; HHA: hip hemiarthroplasty; PHA: partial hip arthroplasty

Year	HA*	THA*	Fracture*	PHA*	HHA/Fracture	Fracture/THA
2004	13.18	9.755	5.64	3.42	0.607	0.427
2005	17.15	12.97	7.11	4.18	0.587	0.414
2006	18.19	13.34	7.03	4.84	0.688	0.386
2007	24.05	19.13	8.25	4.91	0.595	0.343
2008	24.32	19.84	8.52	4.47	0.525	0.350
2009	29.58	25.15	9.53	4.42	0.463	0.322
2010	28.21	23.77	9.79	4.43	0.453	0.347
2011	43.71	38.64	10.14	5.07	0.499	0.232
2012	38.17	33.01	10.91	5.15	0.472	0.285
2013	37.99	33.34	10.83	4.64	0.428	0.285
2014	37.56	33.09	11.06	4.46	0.403	0.294
2015	42.39	37.41	12.04	4.97	0.413	0.284
2016	45.50	40.59	12.68	4.90	0.387	0.278
2017	50.78	46.06	13.47	4.72	0.350	0.265
2018	49.58	45.29	12.59	4.29	0.340	0.253
2019	54.49	50.20	13.16	4.29	0.326	0.246

**Figure 2:**
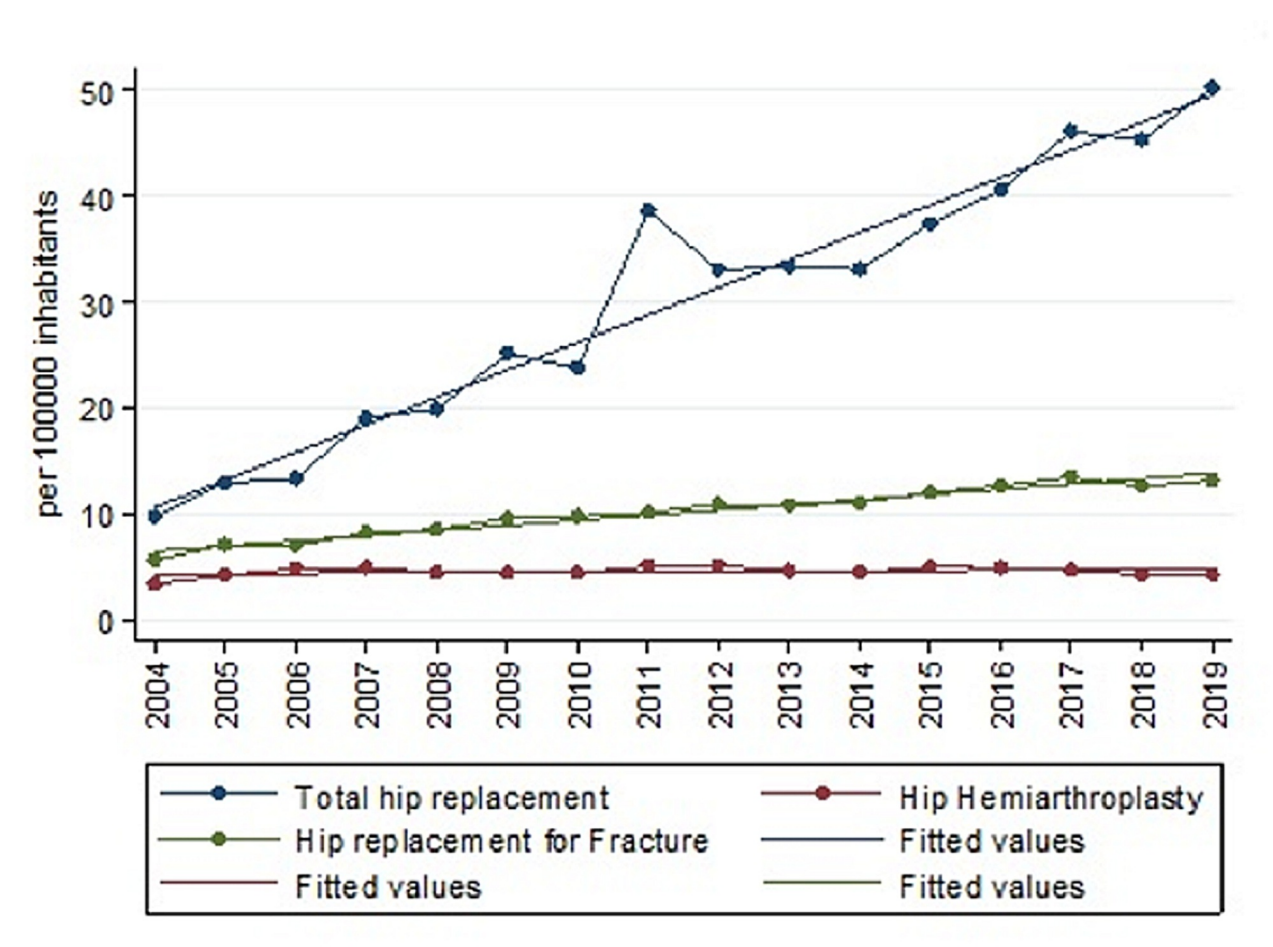
The trend of procedures per 100,000 inhabitants between 2004 and 2019 in THA, PHA, and HA due to intracapsular fractures Abbreviations: HA: hip arthroplasty; THA: total hip arthroplasty; PHA: partial hip arthroplasty

Regarding total hip arthroplasty (THA), the IR in 2004 was 9.75; meanwhile, it was 50.20 in 2019 (Table 1). This upward trend was significant (rho=0.96, p<0.000) and follows a linear model (R2=0.94) of parameters β0=-5192.878 (p<0.00) β1=2.597 (p<0.00), which predicts an IR of 78.14 in 2030 (Figure 2).

**Figure 3:**
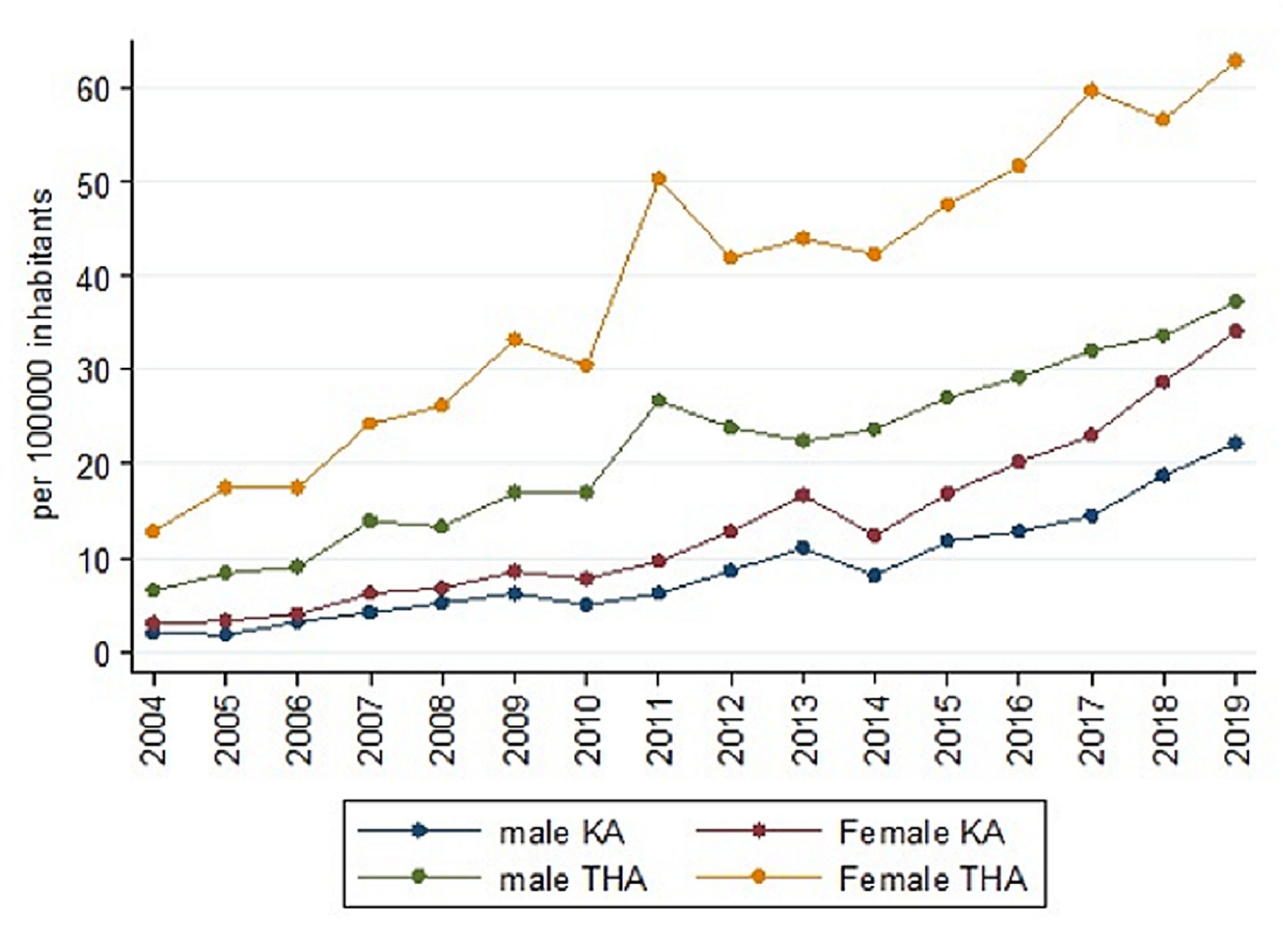
Trend of procedures by gender per 100,000 inhabitants between 2004 and 2019 Abbreviations: KA: knee arthroplasty; THA: total hip arthroplasty

In 2004, the IR in female patients was 12.86, which increased to 62.87 in 2019. This trend was significant (rho=0.9676, p<0.000). Male IR was 37.0 in 2019 (Figure 3) and had a significant upward trend (rho=0.9618, p<0.000). The proportion of males undergoing THA has slightly increased, reaching statistical significance (rho=0.65, p=0.0064) (Table 3).

**Table 3:** Summary by gender of the rate by 100,000 inhabitants of knee and hip arthroplasty *Per 100,000 inhabitants Abbreviations: KA: knee arthroplasty; THA: total hip arthroplasty

Year	Male KA*	Female KA*	Male THA*	Female THA*	Male/KA	Male/THA
2004	2.04	3.06	6.58	12.86	0.399	0.338
2005	1.89	3.27	8.40	17.44	0.367	0.325
2006	3.16	4.05	9.05	17.56	0.438	0.340
2007	4.23	6.20	13.88	24.28	0.405	0.363
2008	5.19	6.77	13.35	26.21	0.433	0.337
2009	6.24	8.56	16.97	33.18	0.421	0.338
2010	5.01	7.78	16.90	30.51	0.391	0.356
2011	6.24	9.68	26.71	50.33	0.391	0.346
2012	8.65	12.84	23.87	41.97	0.402	0.362
2013	11.09	16.68	22.50	43.97	0.399	0.338
2014	8.13	12.42	23.68	42.30	0.395	0.358
2015	11.72	16.88	27.06	47.55	0.409	0.362
2016	12.87	20.23	29.27	51.68	0.388	0.361
2017	14.51	23.07	32.13	59.71	0.385	0.349
2018	18.82	28.74	33.70	56.66	0.395	0.372
2019	22.24	34.10	37.26	62.87	0.394	0.372

**Figure 4:**
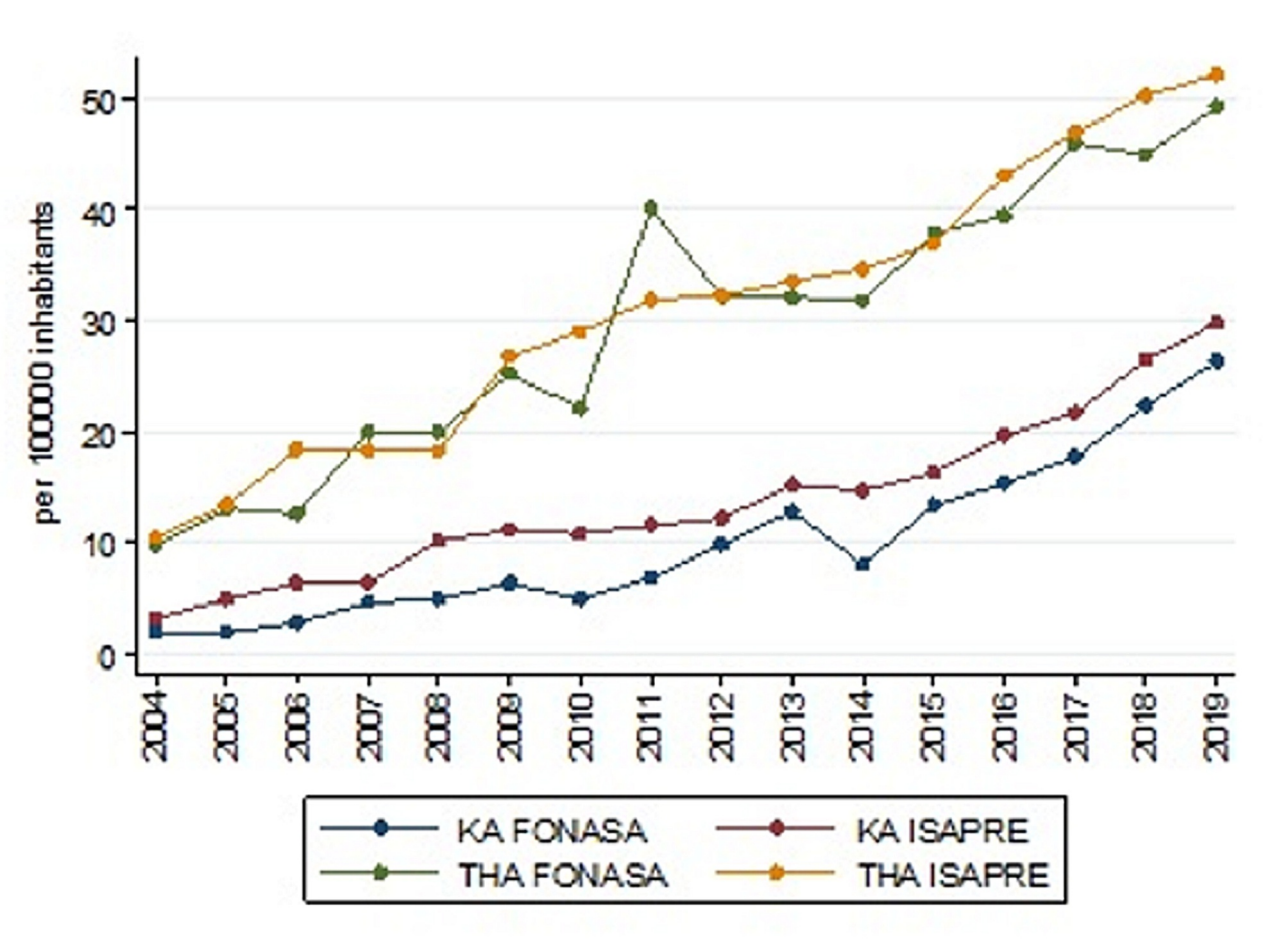
Trend in HA and KA by type of health insurance Abbreviations: KA: knee arthroplasty; HA: hip arthroplasty

**Figure 5:**
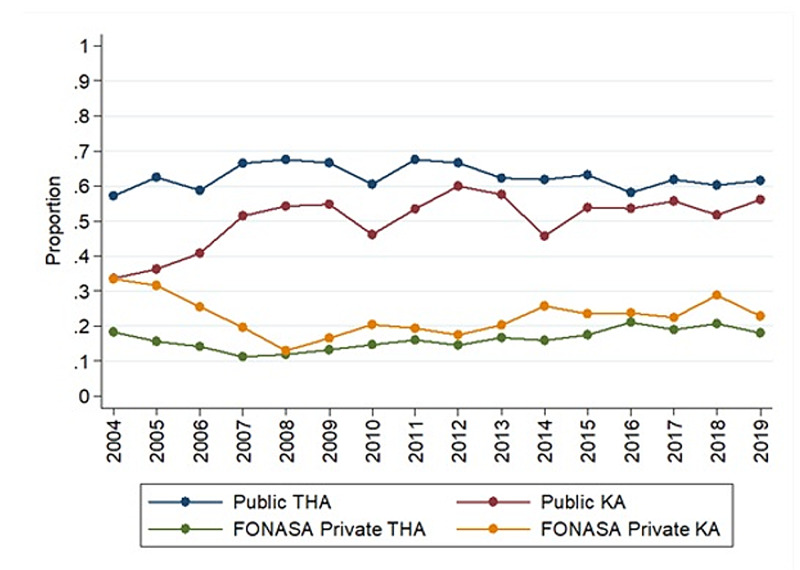
The proportion of patients that underwent HA and KA in a public health center and the rate of patients affiliated to the public insurance (FONASA) that underwent HA and KA in a private health center. Abbreviations: KA: knee arthroplasty; HA: hip arthroplasty

The IR of patients affiliated with FONASA who underwent THA was 9.62 in 2004; meanwhile, it was 49.16 in 2019. However, the IR of patients affiliated with ISAPRE increased to 52.07 in 2019 from 10.4 in 2004 (Figure 4). Between 2009 and 2018, IR in patients above 60 years was 142.25 and 259.29 for FONASA and ISAPRE, respectively - which represents 1.8 HA in patients over 60 years of age affiliated to ISAPRE for every 1 HA of patients affiliated to FONASA over 60 years of age. The proportion of THA performed in PHN increased from 0.57 in 2004 to 0.68 in 2011 (Figure 5); then, it went down to 0.62 in 2019 (rho -0.144, p=0.5944) (Table 4).

**Table 4:** Summary of KA and THA per health insurance and type of institution *Per 100,000 inhabitants Abbreviations: F: FONASA (public insurance); KA: knee arthroplasty; I: ISAPRE (private insurance); THA: total hip arthroplasty; PHN: public health network; PHI: private health institutions

Year	F KA*	I KA*	F THA*	I THA*	PHN/HA	PHN/KA	F PHI KA	F PHI THA
2004	1.87	3.14	9.62	10.40	0.571	0.336	0.334	0.182
2005	1.86	4.86	13.05	13.42	0.625	0.362	0.315	0.155
2006	2.71	6.34	12.54	18.33	0.587	0.408	0.254	0.141
2007	4.58	6.34	19.91	18.28	0.665	0.514	0.196	0.112
2008	4.85	10.11	19.94	18.15	0.675	0.542	0.130	0.118
2009	6.32	11.11	25.20	26.70	0.666	0.547	0.165	0.132
2010	4.84	10.75	22.06	28.94	0.604	0.461	0.203	0.146
2011	6.79	11.52	40.05	31.85	0.675	0.533	0.192	0.160
2012	9.76	12.18	32.11	32.19	0.665	0.599	0.174	0.145
2013	12.80	15.10	32.04	33.52	0.622	0.575	0.202	0.166
2014	8.022	14.68	31.69	34.64	0.618	0.456	0.257	0.158
2015	13.34	16.24	37.78	36.89	0.631	0.538	0.234	0.174
2016	15.27	19.57	39.40	43.00	0.580	0.535	0.237	0.211
2017	17.70	21.62	45.95	46.93	0.618	0.556	0.224	0.189
2018	22.28	26.47	44.87	50.24	0.602	0.516	0.287	0.207
2019	26.28	29.76	49.16	52.07	0.615	0.560	0.228	0.180

In 2004, 42.78% of HA were performed due to hip fracture; meanwhile, in 2019, hip fracture caused 24.16% of the performed HA. This downward trend was significant (rho=-0.87, p<0.000) (Table 1). Of intracapsular fractures, 60.75% were treated with hemiarthroplasty (HHA) in 2004; in contrast, it was only 32.60% in 2019. This downward trend was significant (rho=-0.9588, p<0.000).

Hip revision procedures in 2004 were estimated to be 84 (0.05%); meanwhile, it went up to 341 in 2019 (0.04%). This upward trend was significant (rho=0.96, p<0.000) and follows a linear model (R2=0.91) of parameters β0=-35059.66 (p<0.00) and β1=17.53 (p<0.00), which predict a total of 525 revisions in 2030. The proportion of revision estimated has remained constant (rho=-0.34, p=0.1959).

Knee arthroplasty

A total of 31,943 KA were performed; of them, 17,004 (53.23%) were carried out in the PHN. The median age was 66 years (±11) and 13,512 patients (42.30%) were under 65 years old. Procedures on patients affiliated to FONASA were 21,305 (66.70%); meanwhile, 6,961 (21.79%) were on patients affiliated to ISAPRE.

**Figure 6:**
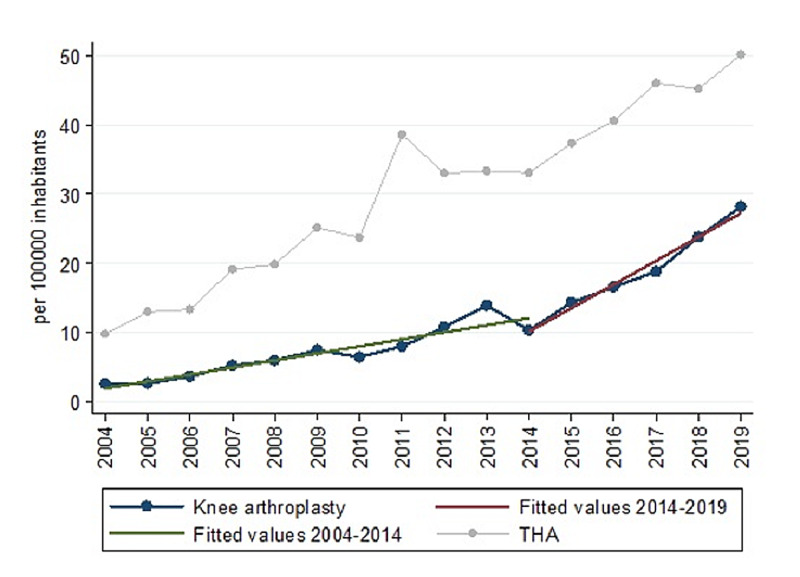
The trend of procedures per 100,000 inhabitants between 2004 and 2019 in KA Abbreviations: KA: knee arthroplasty; THA: total hip arthroplasty

In 2004, KA IR was 2.56, which increased to 28.23 in 2019 (Table 1). This upward trend was significant (rho=0.98, p<0.000). The trend of KA has two slopes, one from 2004 to 2014 and the other between 2014 and 2019 (Figure 6). According to this last slope, a linear regression (r2=0.98) with parameters β0=-6919.51 (p<0.00) β1=3.44 (p<0-00) estimates a rate of 65.14 TKA per 100,000 inhabitants in 2030.

Male IR was 2.04 KA in 2004, which increased to 22.24 in 2019; this trend was significant (rho=0.97, p<0.000). Female IR also had a significant upward trend (rho=0.9794, p<0.000), reaching 26.28 KA in 2019. The proportion of males had remained constant (rho=-0.34, p=0.1919), from 40% in 2004 to 39% in 2019 (Table 3).

FONASA IR was 1.87 in 2004, and it increased to 26.28 in 2019. However, the IR in patients affiliated with ISAPRE increased to 29.76 in 2019 from 3.14 in 2004 (Figure 4). Between 2009 and 2018, IR in patients above 60 years was 54.87 and 137.84 for FONASA and ISAPRE, respectively, which represents 2.5 KA in patients over 60 years of age affiliated to ISAPRE for every 1 KA of patients affiliated to FONASA over 60 years of age. The proportion of patients affiliated with FONASA was 37.3% in 2004 and 47.8% in 2019; this upward trend was statistically significant (rho=0.7441, p=0.0009). In 2004, the proportion of KA performed in the PHN was 33.65%, and it went up to 56.04% in 2019 (Figure 3). This upward trend was significant (rho=0.57, p=0.021) (Table 4).

Knee revision procedures in 2004 were estimated to be 10 (0.02%). Meanwhile, it went up to 105 in 2019 (0.02%). This upward trend was significant (rho=0.89, p<0.000) and follows a linear model (R2=0.75) of parameters β0=-9270.16 β1=4.627, which predict a total of 125 revisions in 2030. The proportion of revision estimated has remained constant (rho=-0.35, p=0.2493).

## Discussion

The main findings of this study were that HA and KA had an upward trend in the period studied and that HA was 2.5 times more frequent per 100,000 inhabitants than KA. Also, female patients underwent a higher proportion of arthroplasty, this difference being wider in hip arthroplasty.

According to a report of OECD, Chile was second last in IR for HA and KA in 2017 [5]. Between 1995 and 2000, Australia had the same population as Chile in 2019 (around 18 million). By then, Australia had an IR between 166 and 179 HA and 181 to 196 KA, way above the 54 and 28 IR found in this study [6]. One factor that is responsible for this difference is the age of the population. According to the World Bank, Europe has 20% of their population above 65 years; Japan has 28%, the United States (US) and Australia have 16%; and the average of OECD countries is 16%; meanwhile, Chile has 12% of its population above 65 years.

Another crucial explanation is that Chile is a country with low coverage for a core set of services and health expenditures (HE). Chile was way below the OECD average of HE estimated in 2018 (3394 USD PPD), reaching 2182 USD per person. HE was 50% government/compulsory and the other half out of the patient’s pocket [5]. Nevertheless, since 2008, Chile has continued to report annual health spending increases above 5%, reaching in 2018, 8.8% of the gross domestic product, which was above the OECD average (8.7%). Also, HE from public sources as a share of total government expenditures in 2017 was 17% (above OECD average 15%) [5], which explains the significant upward trend in IR of arthroplasty.

The Chilean health system relies heavily on PHI [7]. This study reports that near 20% of patients affiliated to FONASA, the public insurance, underwent arthroplasty in a PHI. Also, the proportion of THA in PHN had varied between 58% and 68% between 2004 and 2019. Moreover, PHN had never performed above 60% of the KA, achieving the most in 2013 (59%). In England, an upward trend between 2004 and 2013 was reported in THA in private health centers, going from 2% to 20% [8]. In Australia, approximately two-thirds of the procedures are performed in public centers [9]. Remarkably, between 2009 and 2018, patients above 60 years, who are at a higher risk of needing arthroplasty, represented 17% and 8% of the patients affiliated with ISAPRE and FONASA, respectively [4,10]. Adjusting by age, patients affiliated with private insurance underwent 1.8 HA and 2.5 KA for every one HA and KA undergone by patients over 60 years old affiliated with public insurance. Inequity in access is a significant issue in the Chilean health system [11-12].

Explicit Health Guarantees (GES) are a set of benefits guaranteed by the Chilean law allowing access, opportunity, financial protection, and quality of care in a designated list of diseases. In 2006, HA for patients older than 65 years was included. This is probably the main reason for the different slope found in this study between HA and KA. The 2.6 increment by year is insufficient to reach the IR of OECD countries. Moreover, the Australian and the Finnish registries show that the IR of KA has overcome the IR for HA [13-14]. Regarding knee replacement, GES only covers non-surgical treatment.

The age threshold of GES limits the increase in HA. Reports from National Arthroplasty registers show an increased burden of patients requiring arthroplasty below 65 years of age [15]. Moreover, this study shows that 42% of the patients that underwent HA and KA were younger than 65, leaving them unprotected. Osteoarthritis has the most impact on the US healthcare system due to its high prevalence and disability [16]. Therefore it does not seem cost-effective to exclude working-age patients from GES and condemning them to a waiting list for surgery; especially when THA has been considered the operation of the century due to its impact on quality of life [17]. To add younger patients to GES will allow them to get back to work early. According to unpublished data of the Ministry of Health released by the National Portal of Transparency, the waiting time for surgery between 2012 and 2019 for non-GES HA and KA was 2.19 years (interquartile range, 1.12-3.07). This time is considerably longer than reported by OECD, in which the mean waiting time for HA and KA does not exceed one year [18]. More extensive coverage for HA and the inclusion of KA must be carefully considered in Chilean health policies.

A high proportion of HA replacement has been found in this study due to hip fracture. The reported ratio varies from 2.4% in the US, 4.9% in New Zealand, 6% in Switzerland, to 9.3% in Sweden. Those countries have an IR ranging from 180 to 194 in 2017. Hence the IR for hip fracture is between 4.7 to 16.2, which is like the findings in Chile (13.1). This should focus Chilean efforts to gain access to patients with severe osteoarthritis, which was the main indication (78.9% to 87.1%) for THA in those countries [1].

The optimal treatment for femoral neck fractures in patients over 60 years continues to be debated [19]. The England registry between 2011 to 2016 shows that HHA was used in 50% of neck fractures and THA has a downward trend. United Kingdom's guidelines demand that a hip fracture must undergo surgery in less than three days [20]. Therefore, a possible explanation is that HHA is a more familiar technique for a general trauma surgeon, not requiring to wait for a hip surgeon [21]. On the other side, this study shows a significant downward trend in HHA. No guidelines regarding timing in hip fracture are in Chile, so a more extended hospital stay has been reported [22], which is far from ideal but seems to allow patients to undergo THA. The Australian registry also indicates a low usage of THA in femoral neck fractures, reaching 23.7%; however, the rate was higher when the surgery was performed on weekdays and by a hip surgeon [23]. On the other hand, a report from the US shows that THA for hip fracture has increased in patients below 70 years and patients with private insurance [24].

Arthroplasty nationals registries have contributed to more prolonged survival, better functional outcomes, and the identification of health centers with high rates of complications to improve their results [25]. Chile is having an upward trend in HA and KA but is far behind other OECD countries. Along with improving access, it is time to take the next step in the quality of the Chilean health system and create a national arthroplasty register, aiming to make joint replacements more cost-effective.

The limitations of this study are related to the database. The Chilean national code for surgeries urgently requires an update that allows identifying different subtypes of arthroplasty like unicompartmental knee replacement or revision surgery. Also, the database does not store any variable related to the follow-up of the patients. The strength of this report is that all the procedures performed were included, including the types of institutions or insurance, race, socioeconomic, or geographic factors.

## Conclusions

Arthroplasty represents 0.5% of the hospital discharges between 2004 and 2019 in Chile. Hip and knee arthroplasties were by far the most frequent. Both show an upward trend during the study period with hip replacement being more frequent than knee replacement. Overall, HA was 2.5 times more frequent per 100,000 inhabitants than knee replacement. A significant gap was found in the incidence of arthroplasty with OECD countries, given the less aged population and the inequity in health access that exists in Chile. Wider coverage and a national registry for arthroplasty must be considered in Chilean health policies.
